# The effects of rumination on mood and intrusive memories after exposure to traumatic material: An experimental study

**DOI:** 10.1016/j.jbtep.2009.07.001

**Published:** 2009-12

**Authors:** Ulrike Zetsche, Thomas Ehring, Anke Ehlers

**Affiliations:** King's College London, Institute of Psychiatry, Department of Psychology, De Crespigny Park, London SE5 8AF, UK

**Keywords:** Rumination, Trauma memories, Post-traumatic stress disorder, Self-referent processing, Cognition

## Abstract

Correlational studies have shown that trauma-related rumination predicts chronic post-traumatic stress disorder (PTSD). This study aimed to experimentally test the hypothesis that rumination is causally involved in the development and maintenance of PTSD symptoms. A video depicting the aftermath of serious road traffic accidents was used as an analogue stressor. After having watched the video, *N* = 101 healthy participants were randomly assigned to a guided thinking task designed to induce (a) rumination, (b) memory integration and (c) distraction. In line with the hypotheses, rumination led to less recovery from sad mood triggered by the video than the other two conditions. In addition, self-reported state levels of rumination during the guided thinking task predicted subsequent intrusive memories in the session. However, no significant main effect of the experimental manipulation on intrusive memories of the video was found. Results of exploratory analyses suggested possible sex differences in the way the processing manipulations were effective. Taken together, the results partially support the hypothesis that rumination is involved in the maintenance of negative mood and post-traumatic stress symptoms.

## 1 Introduction

The majority of individuals who experience traumatic events such as a violent assault or severe road traffic accidents report symptoms of distress in the immediate aftermath (e.g. Kleim, Ehlers, & Glucksman, 2007; Murray, Ehlers, & Mayou, 2002). While some trauma survivors then go on to experience persistent symptoms of sufficient severity to warrant a diagnosis of post-traumatic stress disorder (PTSD), a significant proportion recover after a few weeks or months. For example, in the Detroit Area Survey of Trauma 25% of individuals meeting PTSD criteria (except duration criterion) in the weeks after the trauma had recovered at 6 months follow-up whereas 70% still met criteria after a year (Breslau et al., 1998). This raises the question of what factors contribute to the maintenance of the disorder.

A number of theorists have suggested that rumination about the trauma and/or its consequences is an important factor involved in the development and maintenance of post-traumatic stress symptoms (Ehlers & Clark, 2000; Ehlers & Steil, 1995; Joseph, Williams, & Yule, 1995; Wells, 2000). In line with this view, a cross-sectional study of ambulance service workers found rumination to be significantly related to PTSD symptom severity as well as a measure of general mental health (Clohessy & Ehlers, 1999; for similar results see Ehring, Frank, & Ehlers, 2008; Michael, Halligan, Clark, & Ehlers, 2007; Steil & Ehlers, 2000). In addition, a series of prospective longitudinal studies with survivors of road traffic accidents or assaults identified rumination in the immediate aftermath of the trauma as one of the strongest predictors of PTSD symptom severity at 6 months and 1 year, even when initial symptom levels were controlled (Ehlers, Mayou, & Bryant, 1998; Ehring, Ehlers, & Glucksman, 2008; Kleim et al., 2007; Michael et al., 2007; Murray et al., 2002). Interestingly, Michael et al. (2007) found that rumination was not unique to assault survivors with PTSD but was also reported by individuals without the disorder. However, the authors were able to identify specific characteristics of rumination that were closely linked to PTSD severity, namely engagement in “why” and “what if” type questions as well as unproductive circular thinking. In summary, there is evidence suggesting that rumination exacerbates post-traumatic stress symptoms. However, all of the above results are based on correlational data. Therefore, experimental studies are needed to test the hypothesized causal relationship.

Two earlier experimental studies investigated the effect of worry on intrusive images in the context of general anxiety disorder (Butler, Wells, & Dewick, 1995; Wells & Papageorgiou, 1995). In both studies, student participants were presented a distressing film and then asked to either worry about the film and its implications in a verbal form, engage in imagery about the film and its implications, or merely settle down. In both studies, worrying led to significantly more intrusive images about the film than the control conditions. As worry and rumination have been found to share a number of important process characteristics (Ehring & Watkins, 2008), results from these studies can be seen as indirect evidence for a causal effect of rumination on intrusive memories. However, additional analyses in the Butler et al. (1995) study revealed that there were no significant group differences in the time spent worrying during the induction period and that participants in the worry group actually reported more time imaging the content of the film than participants in the imagery or control group. It is thus not clear whether the induction of worry was successful and which processes were responsible for the differences in intrusive memories. In order to clarify the relationship between rumination and PTSD symptoms, studies are needed that induce trauma-related rumination with similar phenomenological properties as those described in PTSD (e.g., Michael et al., 2007).

Little is known about the mechanisms by which rumination exacerbates post-traumatic stress symptoms. It has been suggested that, like worry in generalized anxiety disorder (Borkovec, Shadick, & Hopkins, 1990), rumination might be a form of cognitive avoidance, serving to distract from more distressing cognitions, such as visual memories of the worst moments of the traumatic event (Ehlers & Steil, 1995). This is in line with clinical observations showing that ruminative thoughts often take the form of ‘why’ and ‘what if’ questions rather than focusing on the actual traumatic situation itself. In their cognitive model of PTSD, Ehlers and Clark (2000) suggest three different pathways by which rumination may contribute to PTSD symptoms. First, rumination is thought to prevent an elaboration of the trauma memory and to hinder its integration into preceding and subsequent experiences and other autobiographical memories, which has the consequence that cue-driven retrieval of intrusive trauma memories is not sufficiently inhibited. Secondly, rumination is assumed to strengthen negative appraisals of the trauma and/or its sequelae. Finally, Ehlers and Clark suggest that rumination may also directly trigger symptoms such as feelings of nervous tension, dysphoria and hopelessness, and because it provides internal retrieval cues, may also directly trigger intrusive memories. This proposition is in accordance with recent results from depression research showing that rumination about depressive symptoms exacerbates negative affect, prolongs depressive episodes, and interferes with successful problem solving (Nolen-Hoeksema, 1991; Nolen-Hoeksema & Morrow, 1993; Thomsen, 2006). In addition, Michael et al. (2007) found that negative affect during and after rumination was linked to PTSD severity. The present study therefore aimed to investigate the effect of rumination on negative affect as well as intrusive memories. Since it has been shown that intrusive memories are often triggered by stimuli bearing similar physical characteristics to stimuli signalling the moments of strongest emotional impact (Ehlers et al., 2002), the present study assessed spontaneously occurring memories as well as memories triggered by matching visual and acoustic stimuli.

Effective treatments of PTSD such as trauma-focused cognitive behaviour therapy (TF-CBT) and eye movement desensitization and reprocessing (EMDR) aim to reduce re-experiencing by systematically recounting the traumatic experience or elements of it (see Bisson et al., 2007 for a review). TF-CBT interventions such as repeated imaginal exposure to the trauma (Foa & Rothbaum, 1998) or writing a detailed account of the event (Resick & Schnicke, 1993) aim to systematically transform the fragmented recall of the trauma into a coherent narrative. Similarly, Ehlers and Clark (2000) suggested that in order to reduce re-experiencing, the trauma memory needs to be elaborated. In particular, they suggest that the worst moments of the trauma memory need to be linked in treatment with relevant preceding and subsequent autobiographical experiences that put the meaning of these moments into context (e.g., linking the moment where the person thought that they were never going to see their children again with recent memories of playing with their children that signify to them that they survived the event). In an experimental study, Michael and Ehlers (2007) used a structured writing task to promote such autobiographical memory elaboration and integration after exposure to an analogue stressor. As expected, participants in the memory integration condition subsequently reported fewer intrusive memories than participants in a distraction control condition.

The aim of the present study was to experimentally investigate the effects of rumination on analogue PTSD symptoms, using a distressing video depicting real life footage of the aftermath of road traffic accidents as the stressor. Earlier studies have shown the usefulness and validity of the trauma film paradigm for studying the development and modulation of analogue PTSD symptoms (for a review see Holmes & Bourne, 2008). This study further aimed to extend earlier findings on worry by Butler et al. (1995) and Wells and Papageorgiou (1995) by explicitly inducing trauma-related rumination. Furthermore, the study included two control conditions, a distraction condition, and a condition designed to promote autobiographical memory integration. It was hypothesized that (1) participants in the rumination condition will experience less recovery from sad and fearful mood than participants in the distraction or memory integration conditions, (2) participants in the rumination condition will experience more intrusive memories from the video than participants in the distraction or memory integration conditions, whereas participants in the memory integration condition will experience fewer intrusive memories than those in the control condition, and (3) levels of state rumination across all conditions will be positively associated with the number of intrusive memories.

## 2 Method

### 2.1 Overview

Participants first watched a distressing video showing the aftermath of road traffic accidents (RTAs). The video was followed by a computer-based manipulation of post-exposure processing. Participants were randomly allocated, stratified by sex, to one of three experimental processing conditions: rumination, memory integration, or distraction. The study assessed the effects of the manipulation on mood and intrusive memories during the session and the following week.

### 2.2 Participants

One hundred and one volunteers participated in the study. Exclusion criteria were previous traumatic experiences (including any severe road traffic accidents that participants may have experienced), current depression, current blood/injury phobia, or insufficient knowledge of English to answer questionnaires. Sample characteristics are shown in Table 1.

### 2.3 Materials and tasks

#### 2.3.1 Road traffic accident video

A video comprising eight scenes of real life footage from road traffic accidents (depicting dead bodies, injured people, and emergency personnel at work) served as the analogue ‘traumatic’ stressor. Each scene was briefly introduced by a male voice providing context information. A former version of the RTA video developed by Steil (1997) has been shown to reliably induce negative mood and analogue post-traumatic stress symptoms, such as intrusive memories (e.g. Halligan, Michael, Clark, & Ehlers, 2002; Holmes, Brewin, & Hennessy, 2004). For the current study, some older German footage was replaced with more recent footage shot in the UK. The resulting video lasted 17 min and 30 s and was presented on a 93 × 70 cm TV screen. Participants sat in a comfortable armchair about 2 m from the screen.

#### 2.3.2 Post-video processing manipulation

Participants were randomly assigned to one of three post-video processing conditions, stratified by sex. All conditions had in common that participants were instructed to think about sentences presented on a computer screen. The sentences and their presentation scheme varied according to the experimental condition as specified below.

All post-video processing conditions lasted 12 min and were interrupted at four fixed times when participants were asked to write down some notes about what had been on their minds in the previous interval to allow for assessments of compliance. As a manipulation check, the notes for participants in the rumination and memory integration condition were subsequently rated by two independent raters (who were unaware of the conditions) on four dimensions that have been suggested to be characteristic of ruminative thinking: repetitiveness (defined as repeated thoughts concerned with the same issue; see Ehring & Watkins, 2008), unproductiveness (defined as “why”- and “what if”- questions and other thoughts that are not solution-oriented; see Michael et al., 2007), catastrophizing (defined as thoughts focused on worst case outcomes; Borkovec, Ray, & Stöber, 1998) and abstractness (defined as thinking that is indistinct, cross-situational, equivocal, unclear and aggregated; see Watkins, 2008, for a review of theoretical and empirical work indicating that abstractness is a key dimension distinguishing dysfunctional rumination from functional forms of processing). Each dimension was rated on a 5-point scale from 0 (not at all) to 4 (extremely). Intraclass correlation coefficients computed to establish interrater reliabilities were .75 (repetitiveness), .77 (catastrophizing), .83 (abstractness), and .91 (unproductiveness).

##### 2.3.2.1 Rumination condition

This condition aimed to induce rumination about the video. Participants saw sentences representing ruminative thoughts related to the video on the screen and were instructed to first read the sentence silently and then to think about a concern or a question the sentence might bring up, dwell on its implications, or drift to related issues and follow associated thoughts. Participants were instructed to bring their attention back to the sentence whenever they found themselves thinking about something completely unrelated to the task. They moved on to the next sentence by pressing a button when they felt they had thought enough about a sentence.

The ruminative sentences were chosen to resemble the form (e.g. “why” and “what if” questions) and content of ruminative thoughts reported by RTA survivors with PTSD. For this purpose, a pool of ruminative sentences had been rated by eight therapists specialized in cognitive behavioural treatment of PTSD. Only sentences that had been rated as highly representative of ruminative thoughts typically reported by PTSD patients were included into the task (e.g. ‘Why do people have to drive that recklessly?’, ‘Would I ever be able to be the same person as before?’, ‘How can I drive again without thinking about what could happen?’). A total of 72 ruminative sentences representing 14 trauma-related ruminative topics were selected, with four to seven sentences in each group of topics (see Appendix).

To increase the personal relevance of the rumination sentences, participants were only presented with sentences from topic groups that applied to them. These were identified with a short questionnaire prior to the manipulation task. The questionnaire contained one representative item from each topic group and participants were asked to rate how much each sentence applied to them after having seen the video. For each participant, the seven topics with the highest ratings on the questionnaire were chosen for the rumination task. During the rumination task, the respective rumination sentences were presented on the computer screen one at a time, organized by topic.

##### 2.3.2.2 Memory integration condition

This condition aimed to promote the processing of the film material by asking participants to think about their experience in a self-referential and chronological way. Participants were consecutively presented with seven sets of two to four questions on the computer screen. They were instructed to first read the set of questions silently and then spend the following time thinking about the questions and answering them in their own minds.

The questions were adapted from the writing task used by Michael and Ehlers (2007) to promote autobiographical memory integration by (a) linking the experience of the video to the chronological sequence of events on the same day, and by (b) distinguishing the video from the person's own nontraumatic experiences with road traffic. Specifically, the first set of questions asked participants to think about what they had done and how they had felt before coming to the session. The second set enquired whether the experiment had matched the participant's expectations. The third and fourth set of questions asked them to think back to the video, judge which scenes they found most unpleasant and why and how the scenes were similar or different to own experiences. The fifth set of questions asked participants to think about their own experiences on the road, especially those that they find enjoyable and in which they feel safe. The sixth set of questions asked participants to think about whether they expect the experiment to change their feeling of safety on the road. Finally, the last set of questions asked participants to think about their plans for the rest of the day. Each set of questions was presented for a fixed amount of time.

##### 2.3.2.3 Control condition

This condition aimed to distract participants from thinking about the video while demanding a similar amount of concentration and verbal activity as the other two tasks. Participants were presented with quiz questions on the computer screen asking them to recall as many members of a certain category as possible (e.g. ‘Please try to recall as many African countries/ London boroughs/ US presidents as you can think of’). Participants were instructed to first read each question silently and then spend some time thinking about it and answering it in their own minds. Quiz questions were presented one at a time and remained on the screen until participants proceeded to the next question by pressing the left mouse button.

### 2.4 Dependent variables

#### 2.4.1 Intrusive memories of the video

##### 2.4.1.1 Intrusive Memory Questionnaire (IMQ)

Intrusive memories of the video were assessed with an adapted version of the *IMQ* (Michael & Ehlers, 2007). This questionnaire defines intrusive memories as “*images, sounds or thoughts representing things that were shown, or memories of thoughts/feelings you had while watching the accident scenes*”. Participants are asked to indicate the number of times such memories had popped into their mind during the prior 2 min interval and note this number on the questionnaire. They were instructed not to count any memories they had recalled deliberately.

##### 2.4.1.2 Spontaneous and triggered intrusions during the session

The IMQ was used to assess spontaneous as well as triggered intrusive memories. *Spontaneous intrusive memories* were assessed during two 2 min resting periods; first, after the experimental manipulation task and second, at the very end of the session. The sum of the number of intrusions during these two periods was used as the measure of spontaneous intrusive memories.

The ease with which intrusive memories can be triggered by matching cues was assessed by showing participants a 3 min sequence of stimuli that were taken from the same source of footage as the video, but did not overlap with any part of the video. There were five still images (10 s each) and three acoustic samples (20 s each). All stimuli were separated by a 10 s interval without images or sounds to allow time for possible intrusive memories to emerge. Images were presented on the same 93 × 70 cm screen as the video. Participants reported on the IMQ how many intrusive memories were triggered during the exposure to these probes (*triggered intrusive memories*).

##### 2.4.1.3 Seven day diary

Participants completed a daily diary during the week following the experimental session. The diary comprised seven copies of the IMQ. Participants were asked to fill out one copy each day before going to bed. On the seventh day, participants additionally completed a copy of the PANAS-X (see below). The number of intrusive memories during the week was calculated from the diary entries.

#### 2.4.2 Mood

Parts of the *Positive Affect and Negative Affect Schedule*-*Expanded Form* (*PANAS-X*, Watson & Clark, 1994) assessed participants' mood. Participants are asked to rate 32 adjectives describing mood states on a scale from 1 (very slightly) to 5 (extremely) according to how they are feeling in that particular moment. In order to reduce the number of analyses, only scores for the subscales ‘sad mood’ and ‘fearful mood’ were calculated. The PANAS-X scales have proved to be sensitive to stress, and physical and social activity (Watson & Clark, 1994). In the present sample, internal consistencies were satisfactory; fearful mood: *α* = .68–.89, sad mood: *α* = .75–.85. Participants completed the PANAS-X four times: before and after seeing the video, after the processing manipulation during the session, and again after 7 days.

### 2.5 Additional questionnaire measures

#### 2.5.1 Socio-demographic variables

A seven-item *General Information Questionnaire (GIQ)* was used to assess demographic variables, such as date of birth, gender, ethnic background, first language, marital status, employment status and qualifications.

The *Past Experience Questionnaire (PEQ*; Michael & Ehlers, 2007) was given to screen out subjects with past traumatic experiences, psychotic symptoms, current depression and blood phobia. Furthermore, items assessing usual time watching medical programs and driving frequency were included.

#### 2.5.2 Trait measure of rumination

The 22-item Rumination Scale of the *Response Style Questionnaire* (*RSQ;* Nolen-Hoeksema & Morrow, 1991) was used to check for any pre-manipulation differences in participants' general tendency to ruminate in response to negative mood. Participants are asked to indicate on a scale from 1 (almost never) to 4 (almost always) how often they think or behave in a certain way when they feel depressed (e.g. *Think about how passive and unmotivated I feel*). Previous studies have shown good test-retest reliability and acceptable convergent and predictive validity (Nolen-Hoeksema & Morrow, 1991; Nolen-Hoeksema, Parker, & Larsen, 1994). The internal consistency in this study was *α* = .93.

#### 2.5.3 Depression and anxiety

The *Beck Depression Inventory (BDI;* Beck, Rush, Shaw, & Emery, 1979) is a widely used 21-item self-report measure of depression severity with good reliability and validity (Beck, Steer, & Garbin, 1988). Internal consistency in this study was *α* = .84.

The *State and Trait Anxiety Inventory – Trait subscale (STAI-T;* Spielberger, Gorsuch, Lushene, Vagg, & Jacobs, 1983) is a widely used 20-item self-report measure of trait anxiety that has shown good reliability and validity (Spielberger et al., 1983). Internal consistency in this study was *α* = .92.

#### 2.5.4 Manipulation checks

The *Video Questionnaire (VQ)* comprises one item assessing the distress evoked by the video (rated on a scale from 1 to 100).

The *Processing Manipulation Check Questionnaire (PMCQ)* comprises a total of three items, which were analyzed separately. The first item assesses participants' ability to concentrate on the task by asking how much of the time they were able to think about the questions presented (0–100%). The second item asks participants how distressing they found the task (0 = *not at all* to 100 = *extremely*). The last item assesses the extent to which participants felt driven to continue thinking about the accident scenes (0 = *not at all* to 4 = *very much*).

The *Perseverative Thinking Questionnaire-State Version (PTQ-S)* is a self-report measure of state rumination. The questionnaire was designed by the authors to assess the *process* of rumination independent of its specific content. This is based on the assumption that the negative outcomes of rumination are not only determined by its content, but by the characteristic *style* of thinking. The PTQ comprises 20 items assessing the level of repetitiveness (e.g. *The same thoughts keep coming back to my mind again and again*), unproductiveness (e.g. *My thoughts do not result in any conclusions*) and uncontrollability (e.g. *I can't stop dwelling on certain issues*) of thoughts. In this study, the PTQ was employed to assess state rumination. For this purpose, participants were asked to indicate on a 5-point scale (labelled as *never* to *almost always*) to what extent each statement applied to them *while thinking about the sentences presented on the screen*. The PTQ has been found to possess good psychometric properties (Ehring, 2007). Internal consistency in this study was *α* = .89.

The *Memory Integration Processing Questionnaire (MIPQ)* was developed by the authors to evaluate the degree of elaborative, chronologically structured processing of the film material. It comprises 11 items in the form of statements (e.g. ‘*My thoughts followed a coherent structure*’, ‘*I thought about the accident scenes with reference to my life*’, ‘*I associated the experiment with today's activities*’) that are rated on a scale from 0 (*I never thought this way*) to 4 (*I almost always thought this way*). Internal consistency in this study was *α* = .83.

### 2.6 Procedure

One week prior to the study session, participants received an information sheet and a questionnaire package including the RSQ, BDI, and STAI-T. They were asked to bring the completed questionnaires to the session.

At the beginning of the study session, participants received further information about the purpose and procedure of the study and gave written informed consent. Participants then completed the PEQ and baseline PANAS-X. Next, the RTA video was presented. Following the video, participants' mood was reassessed (PANAS-X). Participants were then allocated to one of the three processing conditions. After completing the experimental manipulation task, the manipulation check questionnaires and the third mood rating (PANAS-X) were administered. Participants then had a 2-min rest period, after which they filled in the first IMQ about spontaneous intrusive memories. Next, the probes for triggering intrusions were presented and participants filled in an IMQ about triggered intrusive memories. After a further 2-min rest period, participants filled in the second IMQ about spontaneous intrusive memories. At the end of the session, the experimenter gave the participants the seven day diary (including the final PANAS-X), which they filled in during the following week and then returned by mail. The response rate for the follow-up questionnaires was 96% (*N* = 97). The experimenter made sure that participants felt well before leaving and encouraged them to contact her if they felt distressed about the experiment in any way. No participant took up this option.

Study sessions lasted 1.5 h on average and participants received £ 20 as a compensation for their time and travel expenses. The study was approved by the local Research Ethics Committee.

### 2.7 Data analyses

Differences between the three conditions on manipulation check measures and intrusive memories were tested with one-way ANOVAs with the factor Condition. Changes in mood were tested with two-way repeated measures ANOVAs with Condition as the between-subjects factor and Time (before versus after video; before versus after experimental manipulation) as the within-subject factor. Significant main effects of Condition were further explored using simple contrasts.

The relationship between processing styles and intrusive memories was assessed by calculating correlations across the entire sample. Due to highly skewed intrusion measures Spearman's correlation coefficient for ranked data (*Spearman's rho*) was used.

All statistical analyses were computed using SPSS version 12.0.1 for Windows and a significance level of *α* = .05 was used in all analyses.

## 3 Results

### 3.1 Baseline group differences

As to be expected with random assignment, participants in the three conditions did not differ in any of the demographic variables or other measures prior to the experimental manipulation (see Table 1).

### 3.2 Manipulation checks

#### 3.2.1 Effects of video on mood

On average, participants rated the video as moderately distressing; *M* = 56, *SD* = 18, on a scale from 0 to 100. Condition did not affect the video-related distress, *F*(2, 98) = .86, *p* = .43, *η^2^* = .02. As shown in Table 2, the video significantly increased PANAS-X ratings of sadness, *F* (1, 97) = 209.2, *p* < .001, *η^2^* = .68, and fear, *F*(1, 97) = 45.5, *p* < .001, *η^2^* = .32. No significant Time × Condition interaction effects were found, showing that participants in the different conditions experienced a similar increase in negative affect, *F*'s(2, 97) < .85, *p* > .43, *η^2^* < .02.

#### 3.2.2 Manipulation checks for experimental manipulation

Results for the manipulation checks are shown in Table 3. Participants in the three conditions did not differ regarding their concentration on the induction task. However, as expected, perceived distress during the manipulation task was significantly lower in the control condition than in the rumination and memory integration conditions, which did not differ from each other. In addition, participants in the rumination group reported a stronger drive to continue dwelling on the video and higher state rumination scores on the PTQ-S than the control group, and, as a trend, than participants in the memory integration condition. For self-reported levels of memory integration processing assessed with the MIPQ, participants in the memory integration condition reported significantly higher levels of memory integration than participants in both the rumination and the control condition.

As expected, independent raters' blind evaluations of the content of thoughts reported in the short breaks during the thinking task showed that participants in the rumination condition thought about the video scenes in a more abstract, unproductive, repetitive, and catastrophizing way, than participants in the memory integration condition (see Table 3).

### 3.3 Main analyses

#### 3.3.1 Effects of the experimental manipulation on mood

Means and standard deviations for sadness and fear at each assessment point are reported in Table 2. There were significant main effects of Time on sadness, *F*(1, 98) = 87.96, *p* < .001, *η^2^* = .47, and fear, *F*(1, 98) = 58.09, *p* < .001, *η^2^* = .37, showing that, on average, negative emotions decreased from post-video to post-manipulation. In line with Hypothesis 1, the main effect of Time on sadness was qualified by a significant Condition × Time interaction, *F*(2, 98) = 5.42, *p* < .01, *η^2^* = .10, which was followed up by separate ANOVAs for each assessment point: Prior to the experimental manipulation, there was no significant effect of Condition on sadness, *F*(2, 98) = .30, *p* = .74, *η^2^* = .01, while there was a significant Condition effect at post-manipulation, *F*(2, 98) = 4.02, *p* < .05, *η^2^* = .08. As expected, simple contrasts showed that participants in the rumination condition reported significantly more sadness than those in the control condition, *p* = .01. There was no significant Condition × Time interaction for fearful mood, *F*(2, 98) = 1.94, *p* = .15, *η^2^* = .04, nor a main effect of Condition, *F*(2, 98) = .08, *p* = .93, *η^2^* = .00.

#### 3.3.2 Effects of the experimental manipulation on intrusive memories during the session

Means and standard deviations for the reported number of spontaneous and triggered intrusive memories are shown in Table 2. In contrast to Hypothesis 2, there were no main effects of Condition on the number of spontaneous or triggered intrusive memories, both *F*'s < .47, both *p*'s > .63, both *η^2^*'s < .01.

In exploratory analyses, the ANOVAs were re-run with Sex as an additional between-subjects factor. Results showed significant Condition × Sex interactions for both spontaneous intrusive memories, *F*(2, 95) = 4.69, *p* < .05, *η^2^* = .09, and triggered intrusive memories, *F*(2, 95) = 7.08, *p* < .01, *η^2^* = .13. Follow-up tests were conducted for male and female participants separately. Results showed that men in the rumination condition experienced significantly more intrusive memories (spontaneous memories: *M* = 7.3, *SD* = 5.13; triggered memories: *M* = 9.9, *SD* = 8.06), than those in the memory integration condition (spontaneous memories: *M* = 2.1, *SD* = 2.97; *p* < .01; triggered memories: *M* = 4.1, *SD* = 3.48, *p* < .05). Furthermore, men in the memory integration condition tended to experience fewer intrusive memories than those in the control condition (spontaneous memories: *M* = 5.5, *SD* = 4.55, *p* < .05; triggered memories: *M* = 7.4, *SD* = 4.43, *p* = .07). Men in the rumination and control conditions did not report different numbers of intrusive memories, *p*'s > .37.

In contrast, women in the memory integration condition reported more triggered intrusive memories (*M* = 10.9, *SD* = 7.67) than those in the rumination (*M* = 6, *SD* = 4.49) or control conditions (*M* = 7.1, *SD* = 6.56), *p's* < .05, which did not differ from each other, *p* = .67. There were no group differences for spontaneous intrusive memories in women, *F*(2, 68) = 1.54, *p* = .22, *η^2^* = .04.

#### 3.3.3 Effects of the experimental manipulation on symptoms in the subsequent week

There were no significant Condition or Time × Condition effects on any dependent variable in the week following the session, all *F*'s < 1, *p*'s > .65, all *η^2^*'s < .03.

#### 3.3.4 Relationship between processing styles and intrusive memories

Table 4 shows the Spearman rank correlations between processing styles during the experimental manipulation and intrusive memories. In line with Hypothesis 3, levels of state rumination across all conditions correlated with the number of spontaneous and triggered intrusive memories in the experimental session. However, there were no significant correlations between self-reports of memory integration processing and intrusive memories in the experimental session.

Exploratory analyses again investigated possible sex differences. Results showed that state rumination was significantly associated with spontaneous intrusive memories in females, *r* = .46, *p* < .001, and triggered intrusive memories in males, *r* = .48, *p* < .001. Unexpectedly, in female participants, but not males, levels of memory integration processing were also *positively* correlated with spontaneous and triggered intrusive memories, both *r's* = .26, both *p's* < .01. Furthermore, memory integration processing and state rumination were unexpectedly *positively* correlated in women, *r* = .28, *p* < .05, but not in men, *r* = −.18, *p* = .33.

Thus, the memory integration processing questionnaire appeared to not specifically assess adaptive processing. In order to generate a measure of memory integration processing that does not involve elements of abstract, repetitive and uncontrollable thinking, z-transformed PTQ-S mean scores were subtracted from z-transformed MIPQ mean scores for the entire sample. As shown in Table 4, memory integration processing adjusted for rumination was negatively correlated with spontaneous intrusive memories in the experimental session. The same pattern was found in the female subsample. In the male subsample, adjusted memory integration processing showed a marginally significant negative correlation with triggered intrusive memories.

## 4 Discussion

The aim of the present study was to experimentally investigate the effects of rumination on mood and PTSD-like symptoms using an analogue design. It was hypothesized that experimentally induced rumination following exposure to a distressing video would result in less recovery from negative affect than the other two conditions. It was further hypothesized that rumination would increase the number of intrusive memories from the video compared to memory integration processing or distraction. In addition, the number of subsequent intrusive memories was expected to be positively associated with levels of state rumination.

The video appeared to be sufficiently distressing to serve as a laboratory analogue for traumatic events as it was rated as distressing by the participants, led to significant increase in negative mood and triggered on average 6.2 spontaneous and 7.8 triggered intrusive memories. Results from the manipulation checks suggested that the experimental post-video processing manipulations were generally successful in generating different mental processes. The control group was clearly distinguishable from the two processing condition groups, and reported less rumination, less memory integration processing, and less distress. Thus, it appeared to effectively distract participants from further processing the distressing material from the video. The two active processing conditions were also distinguishable in that participants in the rumination condition tended to report higher levels of rumination than those in the memory integration condition, and participants in the memory integration condition reported significantly higher levels of memory integration processing than the rumination group.

In line with the first hypothesis, participants in the rumination condition experienced less recovery from sad mood than those in the memory integration and distraction conditions. This finding corresponds to results from depression research showing that rumination in response to depressive mood exacerbates negative affect, which can in turn contribute to the maintenance of depressive episodes (Thomsen, 2006). Results from the present study suggest that PTSD-related rumination exerts similar effects on sad mood, as suggested by Ehlers and Clark (2000). It is noteworthy that this effect was specific to sadness. The lack of a Condition effect for fearful mood might be due to the particular analogue stressor used in this study. The analogue stressor showed the aftermath of road traffic accidents, including a number of fatalities, and did not show accidents while they are happening. As a consequence, participants may have predominantly reacted with sadness and only to a lesser degree with fear. The video produced considerably greater sad mood than fearful mood, although the increase from baseline to post video was highly significant for both mood scales. Future studies will need to investigate the effects of rumination on different mood states with other anxiety-provoking material.

The second hypothesis regarding the effect of rumination on intrusive memories was not supported. No significant effect of Condition on spontaneous or triggered intrusive memories was found. As earlier research has reliably shown sex differences in the frequency and effects of rumination (e.g. Nolen-Hoeksema, 1991), exploratory analyses including sex as an additional factor were conducted and revealed a significant interaction between Condition and Sex. For men, results were largely in line with the hypotheses: Men in the rumination condition experienced significantly more spontaneous and triggered intrusive memories than men in the memory integration condition, and the memory integration condition led to fewer intrusions than the control condition. Thus, processing the video in a way that promoted memory integration appeared to reduce intrusive memories compared to both rumination and distraction in men. However, unexpectedly, the opposite effect was found in women for triggered intrusions: Women in the memory integration condition reported significantly *more* triggered intrusive memories than in the rumination or distraction condition and there was no effect of condition for spontaneous memories. Because of the small number of male participants in each condition, these results need to be interpreted with caution and need to be replicated before any strong conclusions can be drawn. If replicated in future research, two explanations are conceivable: First, the induced processing styles may have different effects in men and women. Second, the memory integration manipulation may not have worked as intended in women. The task was adapted from a writing task with similar questions (Michael & Ehlers, 2007), but the writing component was dropped to make the task comparable to the rumination task. To promote helpful memory elaboration and integration processing it may not be sufficient in all cases to instruct participants just to think about the questions. In addition, some of the questions used in the memory integration condition, for example the question asking why participants found certain scenes of the video particularly distressing, may have directly induced rumination rather than functional processing in rumination-prone individuals. Future research is needed to decide between these alternative explanations.

Consistent with the third hypothesis, correlational analyses showed that the degree of state rumination across all conditions was positively associated with the number of intrusive memories following the experimental manipulation. Thus, there was correlational support for an effect on rumination on subsequent intrusive memories, which is in line with the results of prospective studies showing that rumination after trauma predicts subsequent PTSD symptoms (e.g., Ehring, Ehlers, et al., 2008; Ehring, Frank, et al., 2008, Kleim et al., 2007; Murray et al., 2002). The results extend the earlier studies by Butler et al. (1995) and Wells and Papageorgiou (1995) on worry induction. In contrast to the earlier studies, the content of the rumination induced by the experimental manipulation was modelled on that reported by patients with PTSD. Although levels of memory integration processing were not related to intrusive memories in the total sample, an unexpected positive correlation emerged for female participants only. In order to rule out the contamination of this measure by unproductive ruminative thinking, we computed a new score of adaptive memory integration processing that does not involve elements of abstract, repetitive and uncontrollable thinking. This measure of adaptive memory integration processing adjusted for rumination was negatively associated with intrusive memories in the entire sample, as well as in the female subsample. In sum, the current study provides experimental evidence that trauma-related rumination leads to the maintenance of negative mood as well as correlational evidence showing that intrusive memories are positively correlated with state rumination and negatively correlated with levels of memory integration adjusted for rumination. The results of the experimental manipulation of processing styles on intrusive memories were not supportive of the hypotheses. Results of exploratory analyses suggested possible sex differences in the way the processing manipulations were effective. The finding that the correlational analyses give clearer support to the role of rumination in maintaining intrusive memories than the comparison of the experimental conditions is in line with previous findings (e.g., Halligan et al., 2002). What people actually do during the experimental tasks appears to be more closely related to subsequent symptoms than what the experimenter would like them to do.

A number of limitations are noteworthy. First, although the trauma film paradigm is generally accepted as a valid laboratory analogue for studying processes involved in the development and maintenance of post-traumatic stress symptoms (Holmes & Bourne, 2008), it remains to be shown whether the results generalize to rumination in survivors of real traumas. Second, the fact that the video comprised several scenes from the aftermath of different road traffic accidents may have caused participants to take the perspective of onlookers instead of that of a trauma survivor. The present findings might therefore more closely reflect the cognitive processes in witnesses of traumatic situations than those of individuals directly involved. Third, the reliance on the variables assessed in the session may raise the question whether this study reveals information about the processes involved in the maintenance of the disorder or rather in its development. In future research, the effect of rumination on the maintenance of symptoms could be tested more directly by inducing rumination about the video repeatedly in the hours or days following the session. Another possible limitation of the study concerns the fact that intrusive memories were assessed retrospectively following 2 min resting intervals. Although similar measures have been used in earlier studies (e.g., Ehring, Szeimies, & Schaffrick, 2009; Halligan et al., 2002; Salters-Pedneault, Vine, Mills, Park, & Litz, 2009; Wells & Papageorgiou, 1995), the reliability and validity of this measure is unclear. An alternative way of assessing intrusive memories would be to ask participants to count or signal these memories online as they arise (see Davies & Clark, 1998). While this method avoids the problems related to retrospective assessment, it comes at the cost that the monitoring process may influence the frequency of intrusive memories it aims to measure. Future research should investigate the validity of the different ways of assessing intrusive memories. Lastly, the low number of intrusive memories in the week following the session resulted in restricted power for the analyses of the diary data. In order to increase the number of intrusive memories in the days following the session, future studies may include a second exposure to the reminders of the video after a few days.

Despite these limitations, the results of the current study support the role of trauma-related rumination in the maintenance of post-traumatic stress symptoms. They also offer some support for positive effects of processing that promotes the integration of trauma experiences with other autobiographical memory. More experimental research on these processes, and how they are different, appears necessary.

## Figures and Tables

**Table 1:** Sample description.

		Total (*N* = 101) *M (SD)* or *N(%)*	Rumination (*n* = 32) *M (SD)* or *N(%)*	Memory integration (*n* = 35) *M (SD)* or *N(%)*	Control (*n* = 34) *M (SD)* or *N(%)*	Statistics
Age		24.38 (5.98)	23.69 (4.54)	24.00 (6.14)	25.41 (6.98)	*F* (2, 97) = .79, *p* = .46, *η*^2^ = .02
Sex	Male	30 (29.7%)	8 (25.0%)	12 (34.3%)	10 (29.4%)	*χ^2^* (2,101) = .69, *p* = .71, *η*^2^ = .08
	Female	71 (70.3%)	24 (75.0%)	23 (65.7%)	24 (70.6%)	
Ethnic background	Caucasian	69 (68.3%)	23 (71.9%)	24 (68.6%)	22 (64.7%)	*χ^2^* (2,101) = .39, *p* = .82, *η*^2^ = .06
	Other	32 (31.7%)	9 (28.1%)	11 (31.4%)	12 (35.3%)	

Native speaker	Yes	87 (86.1%)	30 (93.8%)	29 (82.9%)	28 (82.4%)	*χi^2^* (2,101) = 2.28, *p* = .32, *η*^2^ = .15
	No	14 (13.9%)	2 (6.2%)	6 (17.1%)	6 (17.6%)	
Frequency of driving	Never	44 (43.6%)	16 (50.0%)	15 (44.1%)	13 (38.2%)	*χi^2^* (2,101) = 1.72, *p* = .79, *η*^2^ = .13
	<5 times per month	30 (29.7%)	10 (31.3%)	10 (29.4%)	10 (29.4%)	
	>5 times per month	26 (25.8%)	6 (18.7%)	9 (26.5%)	11 (32.4%)	
Hours watching medical TV		5.9 (6.0)	7.2 (7.2)	5.6 (6.0)	5.0 (4.7)	*F* (2,98) = 1.16, *p* = .32, *η*^2^ = .02

Depression (BDI)		4.8 (4.9)	4.8 (5.6)	4.8 (4.8)	4.9 (4.3)	*F* (2,98) = .00, *p* = 1.00, *η*^2^ = .00
Anxiety (STAI-T)		35.7 (9.6)	35.1 (9.4)	35.6 (9.4)	36.4 (10.2)	*F* (2,98) = .14, *p* = .87, *η*^2^ = .00
Trait Rumination (RSQ)		39.1 (11.3)	37.8 (11.7)	39.6 (10.9)	39.8 (11.6)	*F* (2,98) = .32, *p* = .73, *η*^2^ = .01

BDI = Beck Depression Inventory; STAIT = State Trait Anxiety Inventory – Trait version; RSQ = Response Style Questionnaire.

**Table 2:** Means and standard deviations of dependent variables by condition.

		Total (*N* = 101) *M (SD)*	Rumination (*n* = 32) *M (SD)*	Memory integration (*n* = 35) *M (SD)*	Control (*n* = 34) *M (SD)*
*Variables assessed during the session*					
Sadness	1. Pre-video	1.08 (.18)	1.04 (15)	1.09 (17)	1.10 (.22)
	2. Post-video	1.80 (.60)	1.84 (.63)	1.74 (.63)	1.83 (.54)
	3. Post-manipulation	1.38 (.51)	1.58 (.53)	1.34 (.56)	1.24 (.36)

Fear	1. Pre-video	1.23 (.28)	1.20 (.24)	1.25 (.32)	1.23 (.28)
	2. Post-video	1.61 (.66)	1.61 (.74)	1.53 (51)	1.71 (.73)
	3. Post-manipulation	1.27 (.46)	1.35 (.57)	1.25 (.43)	1.22 (.37)

Intrusive memories	1. Spontaneous	6.1 (5.84)	6.4 (5.21)	5.9 (5.48)	6.0 (6.83)
	2. Triggered	7.6 (6.34)	7.0 (5.71)	8.6 (7.27)	7.2 (5.94)

*Variables assessed during the following week*					
Intrusive memories		13.5 (13.90)	14.7 (12.04)	12.9 (14.44)	13.0 (15.26)
Sadness		1.43 (.52)	1.39 (.48)	1.44 (.51)	1.46 (.57)
Fear		1.31 (.46)	1.37 (.61)	1.23 (.27)	1.31 (.43)

**Table 3:** Manipulation check.

	Total (*N* = 101) *M (SD)*	Rumination (*n* = 32) *M (SD)*	Memory integration (*n* = 35) *M (SD)*	Control (*n* = 34) *M (SD)*	Statistics
Concentration on task	84.0 (12.4)	83.3 (11.3)	82.6 (12.9)	86.0 (13.0)	*F*(2, 95) = 1.79, *p* = .17, *η^2^* = .04
Manipulation-related distress	24.9 (23.5)	32.2 (20.8) ^a^	29.7 (25.1) ^a^	13.1 (19.8) ^b^	*F*(2, 95) = 5.48, *p* < .01, *η^2^* = .10
Drive to continue dwelling	2.2 (1.22)	2.5 (1.51) ^a^	2.2 (1.03) ^b^	1.9 (1.04) ^b^	*F*(2, 95) = 3.96, *p* < .05, *η^2^* = .08
PTQ-S	27.8 (12.0)	32.1 (10.7) ^a^	26.9 (12.6) ^b^	24.5 (11.5) ^b^	*F*(2, 95) = 2.73, *p* = .07, *η^2^* = .05
MIPQ	19.9 (7.9)	20.4 (5.2) ^a^	25.4 (7.3) ^b^	13.8 (6.3) ^c^	*F*(2, 95) = 27.51, *p* < .001, *η^2^* = .37
Abstractness^1^		2.89 (.96)	2.07 (.81)		*t* (59) = −3.65, *p* < .01
Unproductiveness^1^		.69 (.92)	.06 (.23)		*t* (63) = −3.90, *p* < .01
Repetitiveness^1^		.52 (.62)	.20 (.38)		*t* (63) = −2.50, *p* < .05
Catastrophizing^1^		.56 (.72)	.17 (.41)		*t* (59) = −2.70, *p* < .05

PTQ-S = Perseverative Thinking Questionnaire – State version; MIPQ = Memory Integration Processing Questionnaire; different superscripts denote differences between conditions at *p* < .05 or *p* < .10 if in parentheses.

1 Blind evaluations of thoughts reported in the short breaks during the thinking task.

**Table 4:** Spearman rank correlations between state processing scores and the number of intrusive memories.

		Spontaneous IM	Triggered IM
State rumination (PTQ-S)	Total	.41**	.25*
	Female	.46**	.13
	Male	.24	.48**

Memory Integration Processing (MIPQ)	Total	.18	.16
	Female	.26*	.26*
	Male	−.07	−.07

Memory Integration Processing, corrected for rumination z(MIPQ) - z(PTQ-S)	Total	−.22*	−.10
	Female	−.25*	.04
	Male	−.17	−.35^+^

* *p* < .05; ** *p* < .01; ^+^*p* < .10. PTQ-S = Perseverative Thinking Questionnaire – State version; MIPQ = Memory Integration Processing Questionnaire; IM = Intrusive Memories.

## Appendix 1

**Ruminative sentences used for the rumination induction**

Topic 1: Anger about existence of terrible events/unfair world

Why are so many accidents happening?

Terrible accidents like these happen every day.

Why is road traffic dangerous?

Why do bad things happen mostly to innocent people?

Why do bad things have to happen?

Why can't we undo such terrible events?

Topic 2: Traffic anxiety: own perspective and victim's perspective

How can I drive again without thinking about what could happen?

How will I feel next time I get into a car?

How will I feel next time I am a passenger?

Could one ever rely on other road users again after experiencing such an accident?

Could one live on, without being scared of what will happen next?

Could one feel confident as a road user again after such an accident?

Topic 3: Worry about dangers to own life

Why can I never be sure that such an accident won't happen to me?

I can never be sure what will happen next.

Bad things can happen at any moment.

I could die at any moment.

My life could be changed for the worse at any moment.

I could experience a bad event at any moment.

Topic 4: Relating video to own life

What if an accident like that happened to my loved ones?

What if that happened to me?

What if this had been my friend?

How must it be to get the message about the sudden death of your loved ones?

How must it be to hear your loved ones scream in pain?

Could I ever stop thinking of those who died in the accident?

Topic 5: Realizing dangerousness

Why have I never thought of what could happen when driving a car?

Why have I never realised that I could die at any moment?

Why have I never considered the fact that something terrible could happen at any moment?

Why have I never considered the fact that my life could be changed for the worse at any moment?

How could I have naively trusted other people giving me a lift in the past?

Why can I never rely on other road users to drive safely.

Topic 6: Perspective of a victim: stroke of fate

How horrible to have your life shattered so suddenly.

It must be awful to have your life suddenly changed for the worse.

One's life can be ruined so quickly.

How would I feel about being crippled for the rest of my life?

Could I ever forgive what has been done to me, after experiencing such an accident?

How would I feel towards the people who did this to me?

How could I ever stop thinking “why me”?

Topic 7: Anger about reckless driving/drivers

Why do people have to drive that recklessly?

Reckless drivers should be punished really severely.

Why should I have to depend on other road users to drive safely?

How can people be that irresponsible, driving to fast when giving someone a lift?

Why are people so reluctant to observe the speed limit?

Topic 8: Perspective of a victim: suffering pain

How slowly must time pass when you are trapped in a car, suffering from excruciating pain!

How would I be able to stand the pain, those injured people suffered?

Will the injured people ever forget the pain they suffered whilst waiting for the paramedics to arrive?

How much pain must the injured people have experienced!

Topic 9: Empathy with victim's pain and disgrace

Could they not have rescued the injured people more quickly?

Why do people have to suffer that much?

Why can't we relieve injured people of their suffering?

How degrading it must be to lie almost naked in the middle of the accident site.

How embarrassing it must be to be in such a state in front of other people.

Topic 10: Consequences of accident for victims

Are the victims going to suffer from permanent disabilities?

Will the victims be able to get over this experience?

What has happened to the people who were injured?

Will the people who were injured ever recover completely?

Topic 11: Perspective of a victim: permanent change

How could I ever deal with such a stroke of fate?

Could I ever get back to normal life after such an experience?

How could I ever become happy again after such an experience?

Would I ever be able to be the same person as before?

Would I ever be able to cope with the experience of such an accident?

Topic 12: Perspective of person responsible for accident: dealing with guilt

Could I ever stop feeling guilty after causing an accident?

Could I ever overcome the fact that I killed someone?

How could I ever stop blaming myself after causing an accident?

Topic 13: Reflecting on own reaction to the video

Has my reaction to the film been adequate?

Why can a simple video upset me?

Why can't I get the moans of those injured people out of my mind?

Why can't I stop thinking about how those injured people must have suffered?

Why do I have to watch such disgusting pictures?

Topic 14: Taking the perspective of a witness/ emergency service personal

How could one possibly become a paramedic, facing disgusting scenes every day?

If I witnessed such an accident, would I still be capable of helping?

How would I react at the site of an accident, seeing all the wounds and puddles of blood?

How would I deal with a real accident if just watching a film has upset me?
